# Predictive factors for polypharmacy among child and adolescent psychiatry inpatients

**DOI:** 10.1186/1745-0179-2-25

**Published:** 2006-09-22

**Authors:** Paul SS Russell, Christina George, Priya Mammen

**Affiliations:** 1 Child and Adolescent Psychiatry Unit, Department of Psychiatry, Christian Medical College, Vellore 632 002, India

## Abstract

**Background:**

Aim was to determine the predictive factors for polypharmacy among inpatient children and adolescents with psychiatric disorders.

**Methods:**

Blinded, case-note review of children and adolescents with ICD 10 diagnosis of psychiatric disorders on psychotropic medication was conducted. Data on demography, illness, and treatment was analyzed with univariate and multivariate techniques.

**Results:**

Proscribing non-pharmacological interventions (OR = 4.7) and *pro re nata *medication (OR = 3.3), increased the risk of polypharmacy. Prescribing physical restraint reduced the risk of receiving multiple medications (OR = 0.3).

**Conclusion:**

Proscribing non-pharmacological interventions, *pro re nata *medication and physical restraints increased polypharmacy.

## Background

Despite the pitfalls of advocating polypharmacy to children and adolescents with psychiatric disorders [1], this practice is alarmingly escalating [2]. This trend is predicted to increase with pharmacological treatments targeting symptoms without a clinical diagnosis, and when a pursuit for treatment perfection with medication is attempted [3]. Polypharmacy lacks safety or efficacy [4], but increases the incidence of drug interactions [5]. There is a need for research on polypharmacy in this population [6]. This study aims to provide further information on the risk factors associated with polypharmacy in the child and adolescent, inpatient, psychiatry population.

## Methods

Subjects included in the study (N = 268) were consecutive admissions to the Child and Adolescent Psychiatry Unit, Christian Medical College and Hospital (a tertiary care centre), Vellore, from January 1997 to September 2001. Children and adolescents below 18 years of age, with an ICD-10 diagnosis (Clinical Guidelines Diagnostic Criteria version) of psychiatric disorder and treated with at least one psychotropic (excluding the anti-parkinsonian medication) were identified from unit registry. Reversible anonymisation as well as restricted access and disclosure of the obtained data ensured the privacy of patients. The Institution's Ethics Committee approved this study. A psychiatrist not part of the treating team reviewed the case-notes for demographic, and illness details (independent variables) made by the treating team during the time of admission prior to the design of this retrospective study. Another psychiatrist blinded to the case note details collected the treatment details from the inpatient admission-discharge records (dependent variable). Only the last admission was considered for subjects who were admitted more than once during this period. There was no discernible change in the prescribing pattern of psychotropics in the unit because of any secular trend, change in institutional protocol or any multiple-drug trial that was underway during this period. Information from these sources was grouped as demographic variables (sex), illness variables (diagnoses, co-morbidity, psychotic symptoms) and treatment variables (*pro re nata *medication, nonpharmacological intervention, physical restraint, side effects). P*ro re nata *was operationalized as medication administered on an 'as and when needed' basis. Nonpharmacological intervention was defined in this study as the psychoeducation about the nature of the illness and supportive psychotherapy administered to the patient and family

Chi Square test, Mann-Whitney U test for comparison between groups and logistic regression (enter method) was used to assess the association of polypharmacy and the three groups of variables. P < 0.05 (two-tailed) was considered significant and data was analyzed with SPSS (version 10.0).

## Results and discussion

103 subjects were included in the study and the rest (N = 165) were excluded, as their psychiatric condition did not require treatment with psychotropic medication (N = 152), or incomplete data set (N = 13). The sample mean (sd) age and number of days spent in the hospital was 14.4 (2.6) years and 34.7 (10.9) days respectively. The mean (sd) age difference in years between the monopharmacy and polypharmacy groups was not statistically significant [14.2(2.7) Vs. 14.7(2.6), Z = -1.05; P = 0.3]. The disorders noted were mood disorders (n = 58), psychoses (n = 33) and others (n = 12). About 49(47.6%) subjects had single medication and 54 (52.4%) had multiple medication where, 35(64.8%) had 2 psychotropic, 13(24.1%) had 3 psychotropic, 5(9.3%) had 4 psychotropic, and 1(1.8%) had 5 psychotropic medications. There was no statistically significant difference in the demographic, illness or treatment variables between groups.

Gender, diagnosis, comorbidity, psychotic symptoms, side effects were not statistically significant in the logistic regression models. Proscribing nonpharmacological treatments was the strongest predictor of risk, and not prescribing *pro re nata *medication also increased the risk for polypharmacy. However, prescribing physical restraint was protective in nature and reduced polpharmacy (Table 1).

**Table 1:** Association between risk factors and polypharmacy

	Mono Pharmacy (n = 49)		Poly Pharmacy (n = 54)		Unadjusted OR	df	P Value	Adjusted OR^a^	95% CI for OR		P Value
	n	%	n	%							
**Demographic**											
*Gender*											
Male	25	24.3	31	30.1	1.2	1	0.5	1.2	0.5	2.7	0.6
Female	24	23.3	23	22.3							
											
**Illness**											
*Diagnosis*											
Psychosis	24	23.3	12	11.7	1.4	1	0.2	1.6	0.8	3.2	0.1
Mood	20	19.4	35	34							
Others	5	4.9	7	6.8							
*Comorbidity*											
Yes	29	28.2	35	34	1.2	1	0.5	1.3	0.6	3.0	0.4
No	20	19.4	19	18.4							
*Psychotic symptom*											
Yes	27	26.2	33	32	1.2	1	0.5	1.2	0.5	2.8	0.5
No	22	21.4	21	20.4							
											
**Treatment**											
*Nonpharmacological*											
Yes	33	32	17	16.5	4.5	1	0.001	4.7	2.0	11.1	0.001
No	16	15.5	37	35.9							
*PRN medication*											
Yes	19	18.7	8	7.8	3.6	1	0.007	3.3	1.2	8.8	0.01
No	30	29.1	46	44.7							
*Physical restraint*											
Yes	39	37.9	32	31.1	0.4	1	0.02	0.3	0.1	0.8	0.02
No	10	20.4	22	40.7							
*Side effect*											
Yes	35	18.4	27	21.4	1.5	1	0.3	1.9	0.7	4.7	0.2
No	19	34	22	26.2							

^a ^Adjusted for age, number of days as inpatient, amount of antipsychotic in chlorpromazine equivalent.

The prevalence of polypharmacy in 52% of the population in this study is less than the 60.3% reported previously [7] and proscription of non-pharmacological therapy and *pro re nata *medication were identified as risk factors for polypharmacy as their odds ratio was high. As the odds ratio was around 1 for most of the other factors, their contribution as a risk or protective factor could not be predicted. However, prescribing physical restraint had low odds ratio suggesting it being a protective factor.

Proscribing nonpharmacological therapies as a risk factor is not surprising as non-pharmacological interventions [8] like psycho education [9,10] when combined with medication have resulted in a more rational use of medication and reduction of polypharmacy in previous studies.

However, in contrast to previous finding, *pro re nata *medication has reduced polypharmacy. This is explained by the treatment policy adopted in the facility, to convert and add *pro re nata *doses (if required on more than two or three occasions) to the existing regular medication regime, resulting in reduced polypharmacy. However, predictive factors documented elsewhere and not included in this study could have affected the results [11].

Beneficial effects of physical restraint reflect the protocol followed in the unit to use physical restraint before chemical restraint because the time spent in restraint is lesser than the elimination half-lives of most of the psychotropics, reducing the risks associated with these medicines [12]. Also, despite the existing negative attitude, the advantages of physical restraint have to be considered in assaultive and destructive children and adolescents [13].

The retrospective nature of the study limited our ability to search for the presence of certain other risk factors of polypharmacy and also it is possible that the small sample size and only those with the most severe psychopathology were on polypharmacy as inpatients compromising the generalizability of the finding.

## Conclusion

In conclusion these results suggest that proscribing non-pharmacological interventions, *pro re nata *medication and physical restraint increases the risk of polypharmacy among children and adolescents with psychiatric disorders. Therefore, prescribing non-pharmacological interventions, *pro re nata *medication and physical restraint might decrease the risk of polypharmacy. However, further studies are needed to validate these findings along with subgroup analysis of multi-class polypharmacy, same-class polypharmacy, adjunctive polypharmacy and augmentation strategies.

## Abbreviations

ICD 10 = International Classification of Diseases, version 10.

PRN = *pro re nata*

## Competing interests

The author(s) declare that they have no competing interests.

## Authors' contributions

PSSR was involved in conception, designing, data analysis and interpretation, drafting and approving the final version. CG was involved in conception, drafting and revising the final draft. PM was involved in conception, drafting and revising the final draft. All authors read and approved the final manuscript.
